# Multiyear Links between Water Chemistry, Algal Chlorophyll, Drought-Flood Regime, and Nutrient Enrichment in a Morphologically Complex Reservoir

**DOI:** 10.3390/ijerph17093139

**Published:** 2020-04-30

**Authors:** Jang HaRa, Usman Atique, Kwang-Guk An

**Affiliations:** Department of Bioscience and Biotechnology, Chungnam National University, Daejeon-34134, Korea; janghara@cni.re.kr (J.H.); physioatique@gmail.com (U.A.)

**Keywords:** dendritic reservoir, monsoon regime, empirical modeling, drought, total phosphorus, chlorophyll

## Abstract

This investigation targeted the largest morphologically complex reservoir (Soyang) in South Korea during 1992–2013. It is a prominent source of domestic water supply, irrigation, flood control, and hydroelectric power generation. Therefore, this investigation focused on regional- to global-scale applications. We revealed the empirical links between chlorophyll (Chl-a) and total nitrogen (TN) and total phosphorus (TP), the impact of the monsoon regime on nutrients, and flood and drought regime. Further, we investigated the trophic status dynamics, tendencies of water chemistry factors, and valuation of zonal water chemistry by the application of a modified multimetric water pollution index (WPI). The physicochemical indicators illustrated significant disparities among the Lacustrine (*Lz*), Transition (*Tz*), and Riverine (*Rz*) zones. The solid contents (TSS) displayed a significant increase in the lake zones in the order of *Lz* (4.58 ± 13.7 mg/L), *Tz* (6.16 ± 16.2 mg/L), and *Rz* (7.38 ± 18.9 mg/L). However, TP and allied chemical species revealed an inverse relationship with the TN:TP ambient ratios. Nevertheless, Chl-a displayed sharp interzonal fluctuations from the *Lz* (2.90 ± 3.29 µg/L) to *Tz* (4.61 ± 4.98 µg/L). The seasonal deviations, however, exposed divergent heterogeneities among the TSS, TN, TP, and Chl-a. The regression plot between the observed and predicted Chl-a in the Soyang reservoir displayed a very strong relationship (R^2^ = 0.997). The seasonal and interannual variations of trophic status displayed a higher impact of precipitation, particularly in the case of TP and Chl-a. The flood years indicated phosphorus limitations, while drought years alluded to the non-algal light limitations (biogenic turbidity). Water temperature (WT), dissolved oxygen (DO), biological oxygen demand (BOD), TSS, TP, and Chl-a displayed decreasing trends in the ambient water. In contrast, pH, chemical oxygen demand (COD), electrical conductivity (EC), and TN displayed increasing tendencies by the application Mann–Kendall trend analysis. The WPI outcomes designated *Lz* with excellent water quality while *Tz* an *Rz* indicated good water quality. It also indicated impending sedimentation tendencies in the *Rz*. In conclusion, our findings indicated fluctuating rainfall patterns (drought and flood conditions) that significantly impacted the Soyang reservoir water quality, flood and drought severity, and trophic status of the reservoir. This study highlights the requirements of further studies to substantiate the drought and flood dynamics and their impacts on nutrients and overall water quality status.

## 1. Introduction

The investigations encompassing nutrients, hydrological variations, drought and flood dynamics, eutrophication, and sustainable water supply are essential to validate the hydrological and ecological functions of large lakes and reservoirs. The number of dams and reservoirs constructed during the 20th century increased rapidly to cope with the growing needs of the burgeoning population [1,2]. With approximately 50.01% of the global freshwater holding capacity [3], the lakes and reservoirs help in flood mitigation and river runoff regulation [4], and control essential hydro-geochemical processes [5,6]. Resultantly, the monitoring of water level, chemistry, flow regime, and nutrient enrichment has become imperative to surmise the water resource availability amid changing climate and serial patterns of drought and flood, and to suffice the growing dependency on built water reserves [7,8,9].

Due to the typical rainfall pattern, several multipurpose reservoirs have been constructed in South Korea. The possible aims include mitigating floods, regulating water storage and supply at a sustainable rate, and hydro-electric power generation [10,11]. The fluctuating rainfall pattern further strengthens the impacts of climate change to induce environmental variations with biological, chemical, physical, and hydrological implications [8,12]. Besides, such environmental shifts triggered by the intense precipitation (flood) and drought cause modifications in the water residence time (WRT) and water budgeting of lakes, along with changing water depth and areal range [13,14]. The artificial lakes created by erecting dams on large rivers generate aquatic ecosystems with characteristic chemical and biological features distinguishing them from natural lakes [15,16]. That is why the inflow patterns strongly influence investigations on artificial lakes, dams, irregular flood discharges, and WRT [17,18].

The summer monsoon impacts the hydrological characteristics to modify the chemical water chemistry, nutrient dynamics, and biological characteristics of the lakes [19,20,21]. The pollutants transported along with run-off currents are gradually transported to the dams, resulting in many pollutants by physical, chemical, and biological reactions [22]. Furthermore, the precipitation pattern and intensity may influence the magnitude of surface and subterranean water flow, primary ions, and dissolved organic matter (DOM) inflows [23,24]. DOM inflows are impacted by flood and drought dynamics, climate change, leaching of nutrients, soil erosion, and microbial transformation [25,26]. The land-use patterns with intensive agricultural activities along with subsequent nutrient leaching, could further enhance the risk of eutrophication [10,27,28,29]. In lakes, phosphorus acts as a limiting factor for biological growth when the loading of phosphorus is not increased artificially [4,11,22,30,31]. Most of the phosphorus inflows to the lake water in the form of dissolved phosphorus [32]. Particularly in intense rain-hit areas, the total suspended solids (TSS) inflow to lakes surges during the monsoon and amplifies the inflow of phosphorus [33,34,35].

Sustainable water quality management has robust implications for fisheries, domestic water supply, hydroelectric power generation, aesthetics, and ecosystem services [2,36,37,38,39]. The seasonal and interannual water quality variations between the lacustrine and riverine zones should be considered when investigating the water quality [4,32]. Phytoplankton are the primary producers in the aquatic food chain and give sensitive responses to the environmental changes in river [40]. The monsoon regime further impacts the nutrient and hydrological regime. However, reservoirs are greatly influenced by the discharging upstream rivers, and phytoplanktonic production might show faster growth rate and slower setting rate. Recent research has reported the impact of upstream inflow is more significant than we estimated. It is further increased due to extremely short WRT (~23 days) plus varied sedimentation rates of planktonic entities [41]. Furthermore, the planktonic life is linked with high turbulence and ample nutrients, while benthic entities are more light-limited. With high levels of nutrients (total phosphorus (TP) > 30 μg/L), planktons could show higher growth because their growth would block the light availability to benthic algae [42,43].

Recognizing the importance of the Soyang reservoir, we planned to see the influence of rainfall patterns, particularly the monsoon regime. We investigated the interannual and seasonal fluctuations in water quality factors, the influence of drought and flood conditions on the productivity and its links with nutrient-contributing factors (total nitrogen (TN), TP, chlorophyll Chl-a). Also, we examined seasonal and zonal variations in nutrient-contributing factors; Chl-a, TN, and TP relationships in three lake zones; variations in trophic state index parameters; trophic state index deviation (TSID); and seasonal trends in the prominent water quality parameters. Furthermore, we designated the water pollution status in the different reservoir zones by the application of a modified multimetric water pollution index (WPI).

## 2. Materials and Methods

### 2.1. Study Area

Soyang Dam is Asia’s most extensive and the world’s fourth-largest rock-filled multipurpose dam, constructed in October 1973 in South Korea. It is the largest and deepest (up to 118 m) human-made lake in South Korea, built on the Soyang River that is the largest tributary of the North Han River watershed [17] that is located near the border between South and North Korea. With watershed area 2703 km^2^, total storage capacity 2900 hm^3^, flood control capacity of 500 hm^3^, and water supply capacity of 1473.1 hm^3^, it stands 123 m high and 530 m long [8].

**Figure 1:**
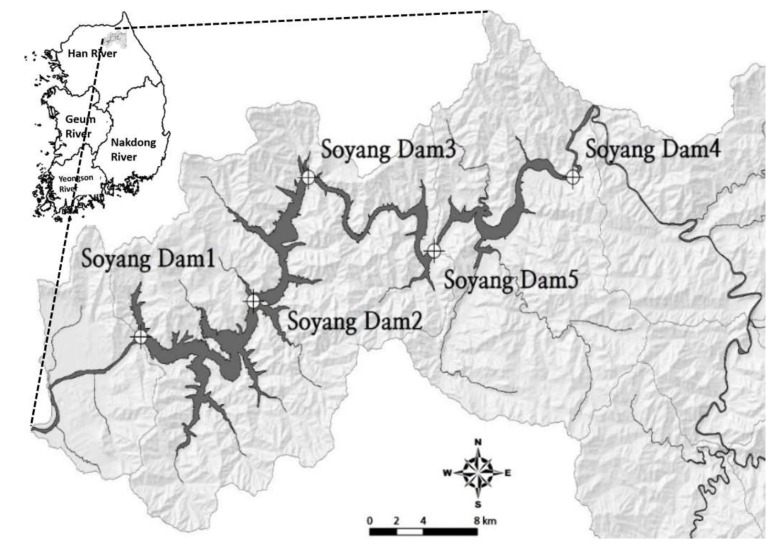
Study area map showing the dendritic-shape Soyang Reservoir, the topography of the reservoir watershed, and the five sampling sites indicated by crossed circles.

With an average annual inflow of 55.5 m^3^/s, the watershed area is subdivided into Yanggu-gun (328 km^2^), Inje-gun (1621 km^2^), Chuncheon-si (349 km^2^), Hongcheon-gun (387 km^2^), and Goseong-gun (18 km^2^). The land-use pattern includes 86% as mountainous-forested terrain (2333 km^2^) in the watershed, while farmland accounts for nearly 6.7% of the total watershed with 178.5 km^2^ land area [44]. This investigation included five study sites in Lake Soyang, and the study area map is presented in Figure 1. The geographic location of each study site in the Soyang reservoir is given in Supplementary Table S1. We divided the lake based on water velocity into three distinct zones, viz., Lacustrine zone (*Lz*, Soyang Dam 1), Transition zone (*Tz*, Soyang Dams 3 and 5), and Riverine zone (*Rz*, Soyang Dam 4).

### 2.2. Analyses of Water Chemistry

The water quality dataset was procured from the Korean Water Environment Information System hosted by the Korean Ministry of Environment. We used a long-term water quality dataset ranging from April 1992 to December 2013 in the three zones of the Soyang reservoir. In total, we investigated 18 water quality parameters. The details of all the water chemistry factors studied are given in Table 1. The Secchi disk (SD) depth was estimated using Secchi disk clarity under the water. The water quality data were obtained every month from each of the designated study sites. The water quality samples were procured in standard sampling bottles from a 50-cm depth of the epilimnetic zone. The sampling bottles were instantly capped to restrict sunlight exposure and stored in an icebox. The pH, DO, water temperature (WT), electrical conductivity (EC), and Chl-a were estimated instantly by employing the multiprobe instrument (YSI Sonde 6600, Environmental monitoring system, Ohio, USA). The TSS, chemical oxygen demand (COD), and BOD were evaluated by the Eaton and Franson [45] method. TN and allied chemical species (NH_4_-N, NO_3_-N, total dissolved nitrogen (TDN)) were chemically analyzed by the second derivative method, and then sample digestion in a persulfate solution was performed [45,46]. TP and related parameters (PO_4_-P, total dissolved phosphorus (TDP)) were estimated by the ascorbic acid method, succeeded by persulfate oxidation [45,47]. Total coliform bacteria (TCB) calculation was performed as per the American Public Health Association (APHA) [48] method. According to the customary procedures, the nutrient-contributing factors (TN, TP) were analyzed in triplicates while BOD, COD, and TSS valuations were accomplished in duplicates [48,49] to ensure dependability.

### 2.3. Precipitation Regime and Flood-Drought Dynamics

The monthly precipitation data were collected from the regional meteorological office. It is essential to state that average annual precipitation in South Korea is higher than the world average rainfall. With a repeated cycle, the average monthly precipitation in January was the lowest at 20.3 mm, while in July, it was recorded as the highest at 383.0 mm. Monsoon (July–August) accounted for approximately 61%, with an average equal to 824.4 mm. The rainfall during the winter months remained very low. It denotes frequent and intensive rainfall episodes in the study area watershed. The designation of a year either as a flood or drought year was established on the basis annual rainfall intensity. If the annual precipitation exceeded 1400 mm, the year was labeled as the flood year, otherwise drought year. The monthly rainfall pattern was used to divide the months as pre-monsoon (January–June), monsoon (July–August), and post-monsoon (September–December).

### 2.4. Establishment of Tropic Status and Nutrient Enrichment

To evaluate the nutrient enrichment and trophic status of the Soyang reservoir, we employed the trophic state index deviation (TSID) in the Lz, Tz, and Rz, as well as studying their seasonal dynamics. The Trophic Status Index (TSI) of the SD (m), TN (mg/L), TP (µg/L), and Chl-a (µg/L) were calculated by using the following equations described by Carlson [50] and Kratzer and Brezonik [51].

TSI (SD) = 60 − 14.41 Ln (SD)(1)

TSI (TN) = 14.43 Ln (TN) + 54.45(2)

TSI (TP) = 14.42 Ln (TP) − 4.15(3)

TSI (CHL) = 30.6 − 9.81 Ln (CHL)(4)

We also applied the Carlson’s trophic state index (CTSI), which is a graphical display of the TSI values of the lakes and reservoirs in an integrated way. The CTSI was calculated by using the following equation:

CTSI = TSI (SD) + TSI (Chl-a) + TSI (TP) ÷ 3(5)

### 2.5. Statistical Analyses

All the datasets were subjected to the Kolmogorov–Smirnov test to check the data normality before performing the log transformations. We performed the data analyses in the context of seasonal (monthly), annual (yearly), and spatial variations (reservoir zones). Further, the seasonal Mann–Kendall Test (MKT) was employed to examine the seasonal trends in the selected water quality parameters, particularly those of concern with respect to human usage [52]. MKT is a nonparametric test to assess the presence of a monotonic trend and is widely used in hydrological time series trend detection. It is a practical approach to planning and management of water resources since it extracts the information on the likelihood of transformation in water quality variables in the future. The MKT assists in calculating the difference between the tendency to increase (denoted by positive sign), decrease (indicated by a negative sign), or no change. Furthermore, we evaluated the zonal water quality status based on salient water chemistry parameters and designated the water quality status of three zones of the lake by following the methodology of Atique and An [4]. For this purpose, we used the modified multimetric water pollution index (WPI). PAST software (Øyvind Hammer, Natural History Museum, University of Oslo, Norway) [53] and Sigma Plot (v. 14.5) (Systat Software Inc., San Jose, California, USA) were utilized as the statistical tools for most of the statistical analyses.

## 3. Results and Discussion

### 3.1. Longitudinal Zonal Dyanmics of Water Chemistry

The survey statistics of the 18 water quality parameters illustrated substantial zonal disparities in Soyang reservoir Lz, Tz, and Rz during 1992–2013 (Table 1). The pH, DO, WT, and EC illustrated an inverse association with the water clarity (measured as SD depth), with an increasing tendency from the *Lz* to *Rz* in the Soyang reservoir. For instance, DO contents in the *Lz* (8.86 ± 2.14 mg/L), *Tz* (9.40 ± 5.00 mg/L), and *Rz* (9.87 ± 2.06 mg/L) with water depths equal to 43.8 ± 32.5 m, 17.4 ± 15.3 m, and 6.00 ± 5.69 m, respectively. Similarly, organic matter indicators (BOD and COD) displayed an increasing trend from the *Lz* to *Rz*. However, interzonal transformations remained insignificant. The TSS showed a significant increase from the *Lz* (4.58 ± 13.7 mg/L), *Tz* (6.16 ± 16.2 mg/L), and *Rz* (7.38 ± 18.9 mg/L) that alluded to the outflow of sediments from the lake regions. The level of TSS corroborated with the levels of ionic substances (measured as EC) as well. The level of TN and related chemical species (NH_4_-N, NO_3_-N, and TDN) displayed an increasing trend from the upstream to downstream zones of the Soyang reservoir with slight or no changes in ammonia nitrogen. However, NO_3_-N indicated the highest mean level in the *Tz* (2.25 ± 27.8 mg/L). On the other hand, the TP and allied chemical species revealed an inverse relationship with the TN:TP ambient ratios. For instance, the highest mean TP level was recorded in the *Rz* (20.7 ± 16.7 µg/L) followed by *Tz* (19.8 ± 23.2 µg/L). However, TDP and PO_4_-P displayed shallow levels in all three lake zones. The TCB levels indicated the Coliform bacteria level in the lake zones and are used as an essential indicator of fecal contaminations [54]. The greater the level of TCB, the higher the chances of fecal pollution in water, with severe dangers of waterborne human infections [55]. The TCB level displayed an increasing trend from the *Lz* (48.13 ± 59.01) to *Rz* (170.6 ± 305.6) during this study. The Chl-a productivity results displayed strong interzonal fluctuation from the *Lz* (2.90 ± 3.29 µg/L) to *Tz* (4.61 ± 4.98 µg/L) while *Rz* Chl-a level (4.55 ± 7.52 µg/L) sustained, like the *Tz* level. The results conformed to the trend of TP, symbolizing thereby inevitable linkages between Chl-a and TP in the three zones of the Soyang reservoir. Before-mentioned interzonal trends and variations indicated the strong association between the nutrient-contributing factors and primary productivity in the lake’s regions. The overall water quality trends indicated that lake water quality was in excellent condition in the *Lz* as compared to the other two zones that showed comparable levels in most of the water quality parameters. The Soyang reservoir water quality summary showed that there were no significant threats of water quality deterioration during the study duration. However, it is essential to consider that the anthropogenic impacts are on the surge that could lead to an imminent water quality crisis.

### 3.2. Influence of Precipitation on Seasonal and Interannual Water Chemistry

**Figure 2:**
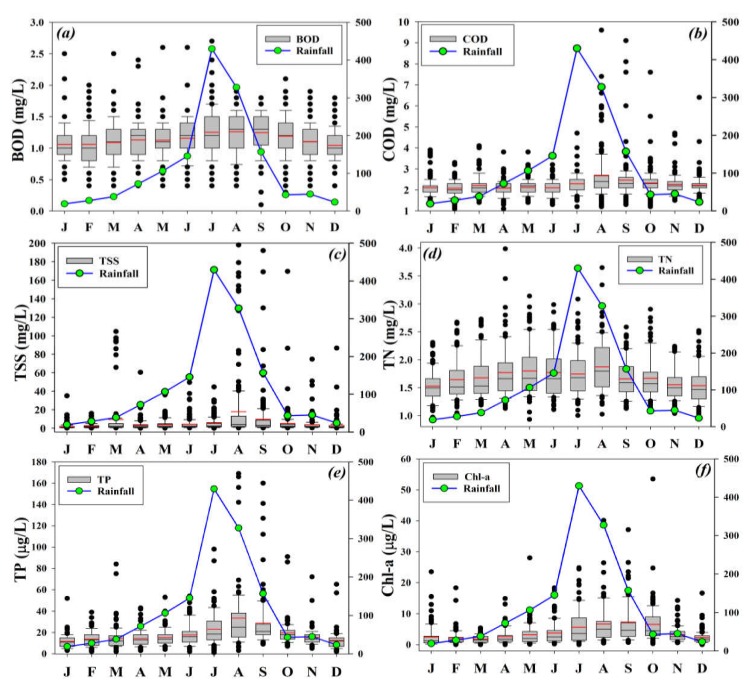
Monthly variabilities of the selected water chemistry parameters (**a**) BOD, (**b**) COD, (**c**) TSS, (**d**) TN, (**e**) TP, (**f**) Chl-a, in relation to seasonal rainfall (mm) intensity during 1992–2013. The right-hand y-axis indicates rainfall (mm).

The seasonal deviations in organic matter, suspended solids, nutrients (TN, TP), and primary productivity indicator (Chl-a) exposed divergent heterogeneities under the influence of the rainfall regime (Figure 2). The results alluded to the increasing inflow of oxygen-demanding chemicals that could have been generated by the variety of anthropogenic and geochemical activities in the lake watershed. However, the mean seasonal variations of BOD and COD demonstrated a similar pattern with a slight peak in corroboration with the monsoon peak. The TSS showed high inflow events linked with intensive monsoon rainfall. The TN levels increased with the intensity of seasonal rainfall and touched the highest mean TN level in August that received the highest rainfall, crossing 430 mm. The TP level, however, exhibited a remarkable increase during the monsoon season and reached up to 170 µg/L. Although Chl-a showed a similar trend as that of TP, it is noticeable that where the Chl-a level approximated the rainfall intensity, it showed a gradual decline in the post-monsoon months. It showed that the lake water was substantially enriched with the nutrients, especially TP that made up the primary production even at shallow precipitation events.

**Figure 3:**
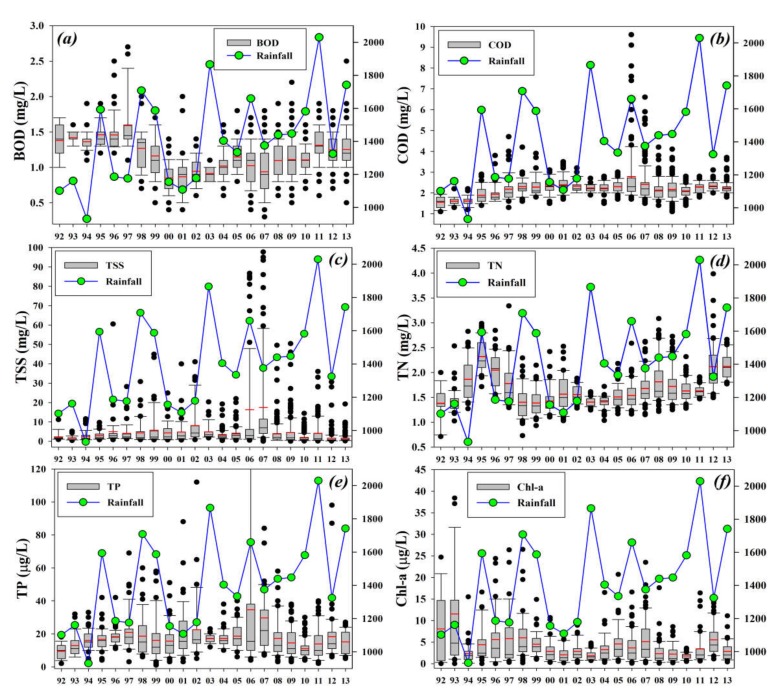
Interannual fluctuations in the selected water chemistry parameters (**a**) BOD, (**b**) COD, (**c**) TSS, (**d**) TN, (**e**) TP, (**f**) Chl-a, with annual rainfall (mm) intensity in the Soyang reservoir watershed during 1992–2013. The right-hand y-axis indicates rainfall intensity (mm).

The watershed received varying rainfall intensity during the study, and it showed ample impact on the annual variations of the selected water quality parameters in the Soyang reservoir (Figure 3). We designated specific years as flood (>1400 mm per annum) and drought years based on rainfall intensity. Here we discuss the annual variations of some water chemistry parameters with the rainfall intensity. For instance, BOD showed an inverse relationship with rainfall during 1992–2013 by showing a decline during drought years and vice versa, while COD showed a gradual increase despite many flood years. It alluded to the persistence of oxygen-demanding chemicals in the lake water that entered the lake water and did not move out. It is also significantly important to mention that a consistent increase of chemical pollutants in lentic water bodies poses grave threats to the intended usages of the water resource.

TSS showed mixed responses to the varying intensities of annual precipitation. TN, however, showed varied responses, seldom approximating and other times opposite to the annual rainfall curve. This indicated the intensity of nitrogen contributing anthropogenic activities remained fluctuating during the study duration. The interannual TP and Chl-a levels showed similar relationships with the rainfall intensity. The mean TP levels increased during the flood years and declined during the drought years. Similar was the case with Chl-a productivity during most of the years. The seasonal and annual variabilities in the featuring water chemistry factors displayed distinct heterogeneities in the oxygen-demanding chemicals and nutrients. The remarkably higher loadings of TSS, TN, and TP provided insights into the single maximum rainfall episodes during the study duration. The flood years mostly indicated an increase in the nutrient-contributing parameters, while drought events caused a decline. It could be inferred that the drought events helped to reduce the nutrient loads in the lake water [2,4,14].

The rainfall acted as a connecting tool between the lake water and anthropogenic activities in the watershed that later showed considerable fluctuations in the critical water quality factors. Some of the preeminent reasons for seasonal and annual water quality variations other than rainfall intensity could be the uncontrolled wastewater discharges, intermittent river inputs, and surface runoff that could be the likely sources of high nutrient-contributing factors in Soyang reservoir. Our findings corroborated several previous studies at the regional and global scale [20,22,39,56].

### 3.3. Spatio-Seasonal Comparisons between TN, TP, and Chl-a

**Figure 4:**
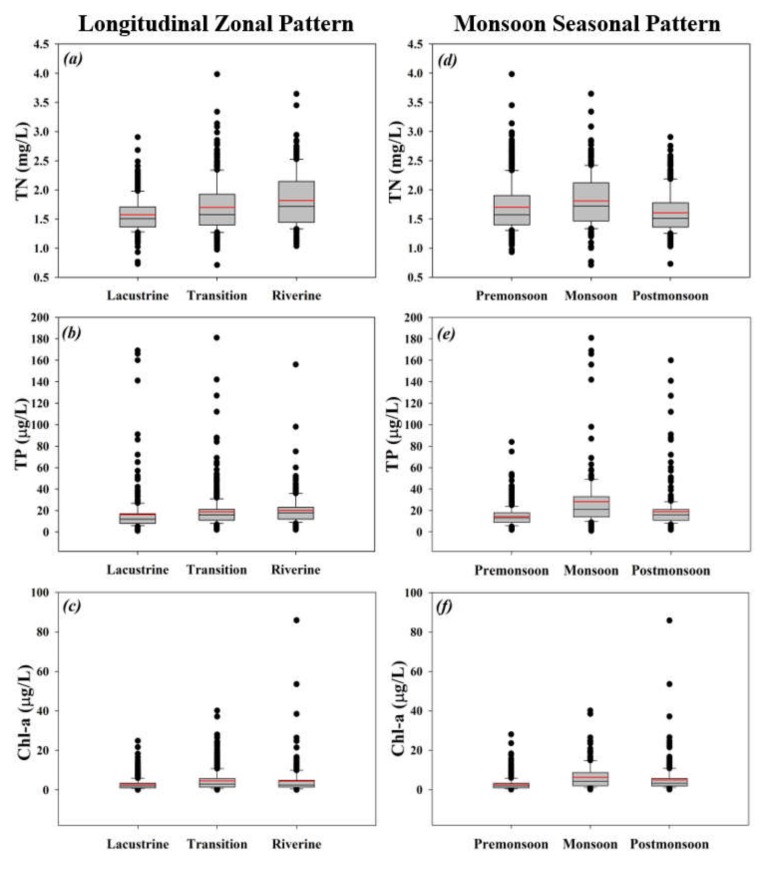
Comparisons between longitudinal zonal and seasonal monsoon patterns of TN, TP, and Chl-a in the Soyang reservoir during 1992–2013. Where (**a**), (**b**) and (**c**) show the longitudinal zonal patterns if TN, TP and Chl-a, respectively, while (**d**), (**e**) and (**f**) indicate the seasonal monsoon patterns of TN, TP and Chl-a, respectively.

We used the box-plot approach to assess the discrepancies between TN, TP, and Chl-a at the spatial (*Lz, Tz,* and *Rz*) and seasonal (pre-monsoon, monsoon, and post-monsoon) scales during 1992–2013 and the results symbolized a diversity in the establishment of the nutrient-contributing factors in Soyang reservoir (Figure 4). At the spatial scale, TN and TP mean values displayed an increasing trend from the *Lz* to Rz, while Chl-a did not indicate significant fluctuations in the lake zones. The growing trend could be associated with the high sedimentation rates in the *Tz* and *Rz* as sediments adsorb the TN and TP particles and serve as a sink, which led to a consistent increase [17,57].

The transport of phosphorus into the aquatic ecosystems comes from the surrounding terrestrial landscapes and natural resources in particulate and dissolved states. Therefore, the sediment acts as the primary sink of phosphorus due to the higher affinity of phosphorus to soil particles [58]. Besides, lake hydrodynamic patterns support the internal loading of phosphorus and nitrogen, which might be the leading reason for approximately 12% of global river phosphorus retention in lake ecosystems [59]. On the other hand, however, seasonal variations appeared to be dominated by the intensive monsoon rainfall. TN, TP, and Chl-a displayed a distinct mean increase during the monsoon season that declined in the ensuing months in all cases. However, the seasonal difference was lower in Chl-a and higher in TP.

### 3.4. Spatio-Seasonal Empirical Modelling of Nutrients and Chl-a

**Figure 5:**
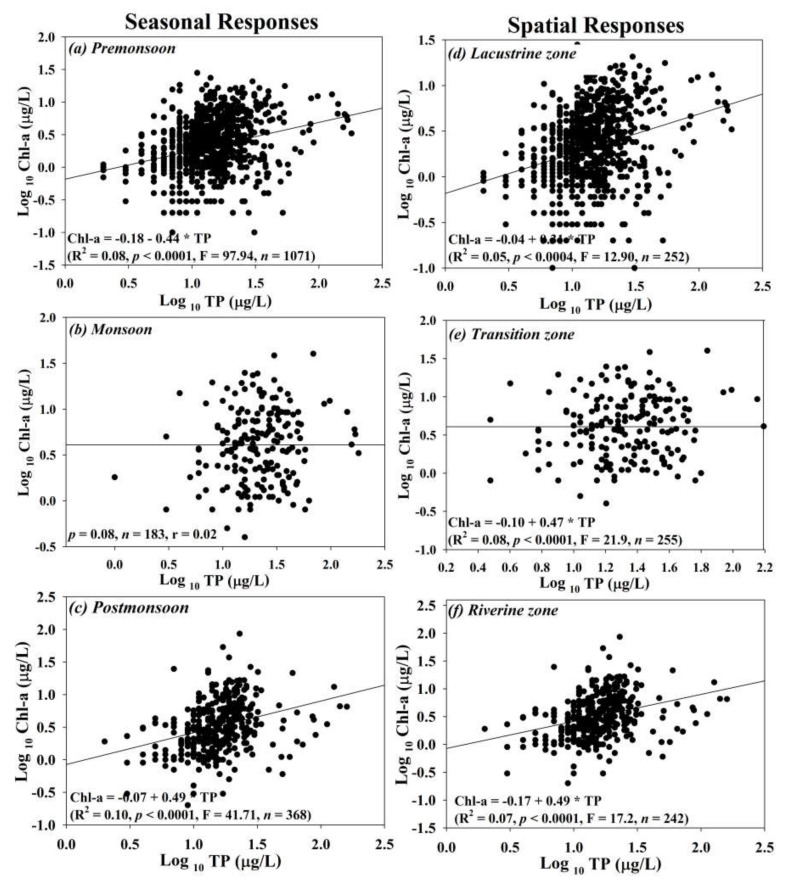
Seasonal (**a**–**c**) and spatial (**d**–**f**) response analyses of Chl-a and TP in the Soyang reservoir during 1992–2013; (*)—Multiplication sign.

We studied the regression relationship of Chl-a on TP at the spatial and seasonal scales to ascertain the degree of dependence of Chl-a on the nutrient-contributing factors, particularly TP, and the results showed a weak positive regression relationship (Figure 5). In the seasonal responses to TP, Chl-a indicated very week response ((R^2^ = 0.10, *p* < 0.0001, F = 41.71, *n* = 368) to the fluctuating TP levels during the post-monsoon season while pre-monsoon and monsoon seasons displayed even weaker (hardly any) responses. However, it is essential to state that the links between Chl-a and TP during the monsoon showed a fragile relationship. It could be linked with the decline in the water residence time (WRT) in the lake during intensive rainfall and wearing away of the existing Chl-a. The spatial scale, however, showed fragile relationships in all three zones. The *Tz* showed comparatively higher links between Chl-a and TP (R^2^ = 0.08, *p* < 0.0001, F = 21.9, *n* = 255). The relationships in the three lakes zones exhibited positive relationships.

**Figure 6:**
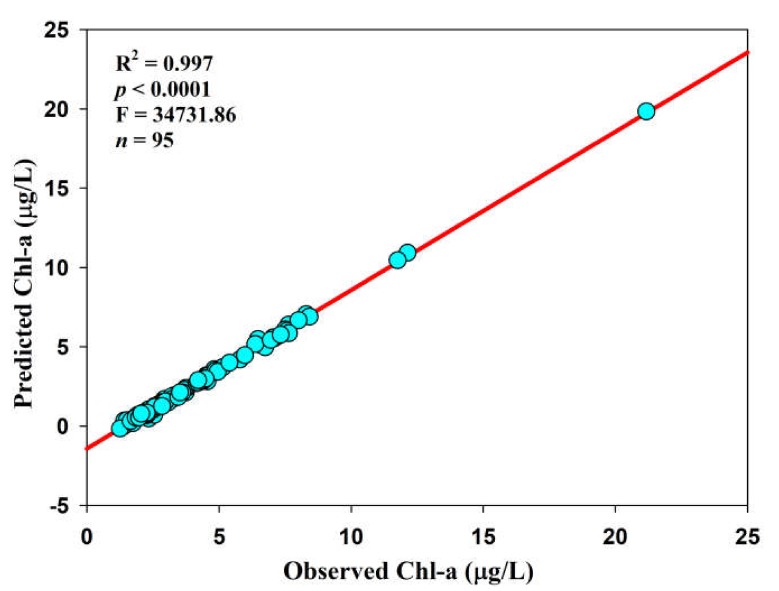
Regression relation and success of Chl-a prediction with observed Chl-a in the Soyang reservoir.

This study does not present the regression analyses between Chl-a and TN on spatial and seasonal scales due to the absence of any trends. Consequently, it is valid to state that TP performed as the limiting nutrient in the Soyang reservoir, like the preponderance of other lakes and reservoirs in South Korea. The primary sources of TP could be linked with agriculture, especially intensive paddy culture and vegetable growing, along with industrial discharges. However, no relationships during monsoon and higher links in the *Tz* allude to the role of longer WRT [60,61,62]. Furthermore, we explained the relationships between observed Chl-a (Obs Chl-a) and predicted Chl-a (Pre Chl-a) by using the regression equation developed by An and Park [21]. The Chl-a level was predicted by using the quadratic relationship of Chl-a = −2.36 + 4.34 log TP − 1.24 (log TP)^2^. The equation of Chl-a developed the regression with TP, and the regression plot between the observed and predicted Chl-a in the Soyang reservoir displayed a powerful regression relationship (R^2^ = 0.997), as shown in Figure 6.

### 3.5. Regression Analyses on Chl-a and Nutrients during Flood and Drought Conditions

**Figure 7:**
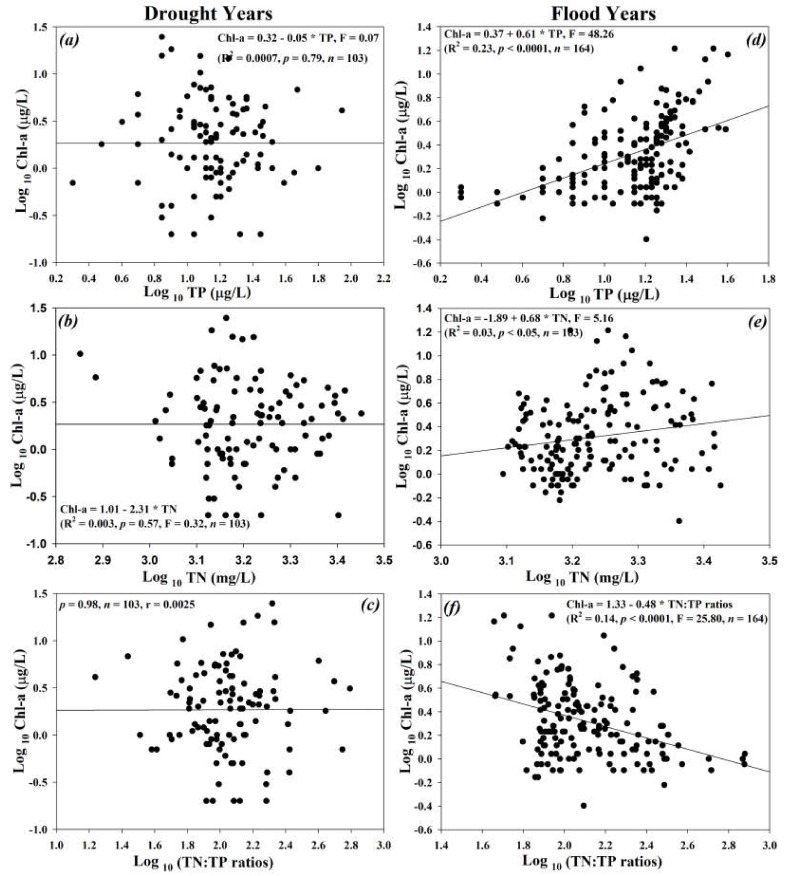
Empirical modeling of Chl-a and nutrients during the flood (**a**–**c**) and drought (**d**–**f**) conditions during 1992–2013 in the Soyang reservoir. (*)—multiplication sign.

The regression analyses on Chl-a with nutrient-contributing agents (TN, TP) and their ambient ratios (TN:TP) showed weak positive regression links between Chl-a and TP (R^2^ = 0.23, *p* < 0.0001, *n* = 164) during flood times, while registering no relationship between Chl-a, nutrients, and their ambient ratios (*p* > 0.01) in the drought conditions (Figure 7). Nevertheless, Chl-a displayed mild negative (R^2^ = 0.14, *p* < 0.0001, *n* = 164) regression correlation with TN:TP ratios in flood years. Fluctuations between flood and drought states may have higher impacts on the chemical water quality, especially primary productivity in lakes and reservoirs. The water chemistry fluctuations become even more vital in the case of rain-fed reservoirs (i.e., influenced by intensive monsoonal rainfall). The spatiotemporal imbalance of water resource distribution led to flood and drought conditions [63]. During recent decades, the impact of changing climate and intensifying anthropogenic actions have transformed the uniform functions of the hydro-meteorological regime, i.e., rainfall and runoff [64]. Such a deviation, coupled with anthropogenic activities and local events of high temperature, has increased the vulnerability of lakes and reservoirs to frequent flooding and drought conditions [7]. With the advent of time, societal dependence on lakes is at risk due to regular imbalance in hydrological regimes. The primary productivity and nutrient-contributing factors (TN, TP) are largely linked with each other. Moreover, the drought and flood conditions could also topple down the WRT, steady inflow and outflow of nutrients, and enhance water scarcity, physical damages to the installations, and decline of biodiversity. The investigations into the drought and flood conditions are more relevant in the case of the Soyang reservoir due to the larger human population dependence on water abstraction, aesthetic value, a resource of aquatic species, and hydropower generation. Recently, it has been noticed that South Korea is at a greater risk of rapidly happening climate change and its devastating impacts. These could be traced in the form of evident fluctuations in the monsoon rainfall patterns and intensity along with long records of air temperature events during the recent years. Therefore, research on drought and flood occurrence and their concomitant impacts is becoming inevitable, particularly in the larger reservoirs such as the Soyang reservoir.

### 3.6. Dynamics of Trophic Status

#### 3.6.1. Seasonal and Annual Trophic State

**Figure 8:**
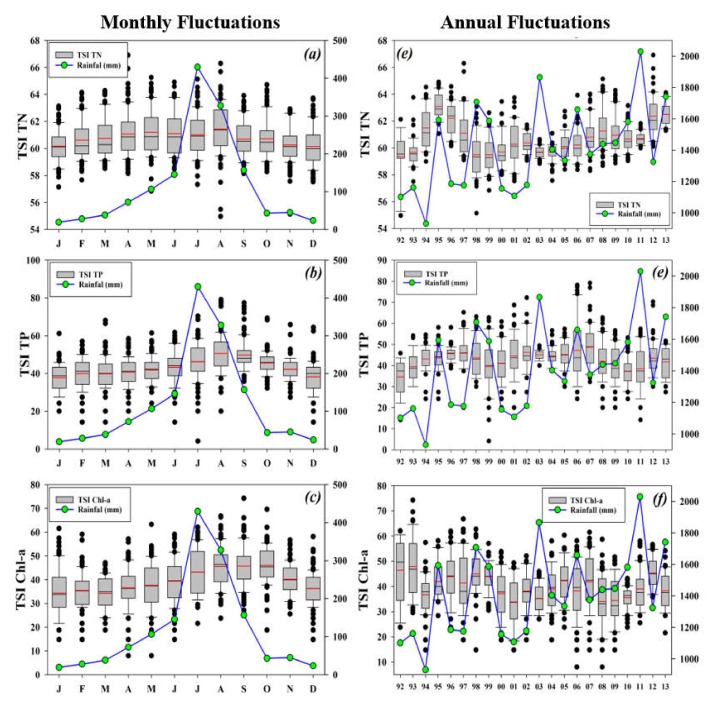
Seasonal (**a**–**c**) and interannual (**d**–**f**) fluctuations in the trophic state index dynamics in the Soyang reservoir. The right-hand y-axis indicates rainfall intensity (mm).

The seasonal and interannual variations of nutrients’ trophic status presented a strong impact of precipitation, particularly in the case of TP and Chl-a (Figure 8). TSI (TN) exhibited an exact approximation with the seasonal rainfall curve in the Soyang reservoir watershed. However, the mean TSI on TP and Chl-a showed similar values and responses to the monsoon rainfall. The interannual variations of TSI (TN) tightly linked with the annual rainfall fluctuations. The TSI (TN) dropped down during the drought while increasing during the flood years. However, the TSI (TP) and TSI (Chl-a) did not show rigorous approximations with annual rainfall. For instance, TSI (TP) revealed an even increase from 1992–1997 and then displayed a decline during 1998–1999, although these were the flood years in the Soyang reservoir watershed. It indicated the greater impacts of rainfall on the trophic state indicators, particularly TP. The year 2007 showed the highest trophic state in TP, which happened to be after a preceding flood year (2006). Therefore, we inferred that the intense rainfall, specifically during the monsoon period, could transport higher levels of nutrients (particularly TP) into the lake ecosystems that are mainly mixed with the inflowing rapid water inflows. These new additions of the TP are expected to be retained in the system for longer times [4], have been limited, and the nutrients might have sustained a balanced trophic status.

The evaluations of TSI based on monsoon regime (pre-monsoon, monsoon, and post-monsoon) demonstrated the crucial role of monsoon rainfall (Table 2). TSI (TN) displayed the same trophic status (TSI (TN) = 61) during all the seasons, while monsoon showed a marked increase in the trophic status in the case of (TSI (TP) = 52) and Chl-a (TSI (Chl-a) = 48). The increase in trophic status during and post-monsoon times further established the role of TP as the limiting nutrient in the Soyang reservoir. The Soyang reservoir manifested high-oligotrophic to the low-mesotrophic state during the pre-monsoon period, while it exposed high-mesotrophic to low-eutrophic status (TSI (TP) = 52 and TSI (Chl-a) = 48) during the monsoon months. It showed a higher dependency of Chl-a on TP for primary productivity. Lentic waters’ eutrophication has emerged as one of the most severe threats to the effective management of water resources [28,65]. Several lakes undergo water quality deteriorations due to the negative influences of nutrient inputs, turbidity, harmful algal blooms, and drought conditions, thereby further restricting their role in local socio-economic development. Therefore, predictions of imminent eutrophication events may provide useful insights into the global- and regional-scale trends in lake water chemistry and ecosystem health management.

#### 3.6.2. Trophic State Index Deviation (TSID) Analyses

**Figure 9:**
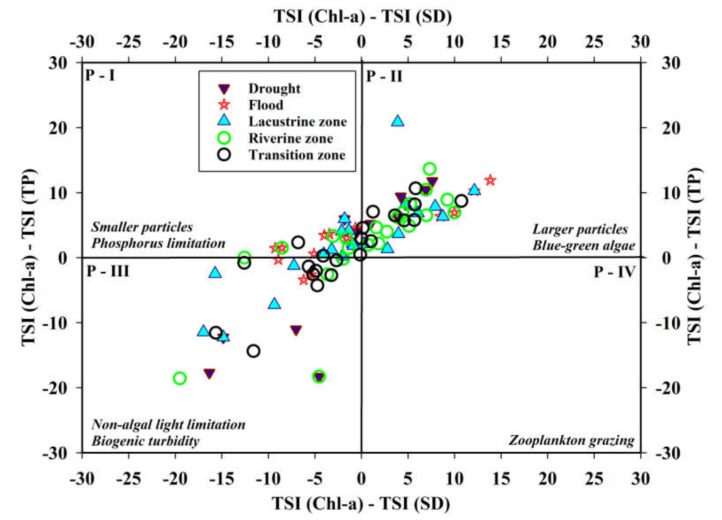
Trophic state index deviation graphical display during flood and drought conditions and in different reservoir zones.

We plotted the trophic state indices to understand their potential deviations at the spatial levels and under drought and flood conditions in the Soyang reservoir. The two-dimensional graphical representation of the nutrient (TSI (TP)), primary productivity (TSI(Chl-a)), and underwater visibility (TSI (SD)) based on Carlson’s [50] research indicated that the trophic deviations revealed a miscellaneous response (Figure 9). We performed the TSID investigations on spatial (*Lz, Tz*, and *Rz*) and rainfall regimes (flood and drought) during 1992–2013. The preponderance of yearly observations settled in the larger particles’ compartments. The flood years indicated phosphorus limitations, while drought years alluded to the non-algal light limitations (biogenic turbidity) in the Soyang reservoir. It is important to specify that the zooplankton grazing was not manifested during this study. The *Lz* exhibited partial phosphorus limitation and infrequently alluded to biogenetic turbidity. The escalating appearance of larger particles and blue-green algae during the drought years and in the *Lz* and *Rz* with the lower tendencies of phosphorus limitation and biogenic turbidity indicated higher possibilities of eutrophication events and nutrient enrichment. Further, during the drought periods, the presence of blue-green algae could be associated with longer WRT and reduced water levels. Despite high-nutrient and sediment-rich inflow, the previous eutrophication events would have been limited, and the nutrients might have sustained a balanced trophic status.

However, unreciprocated manifestations in annual TSI levels of TP and Chl-a suggested that the lake is retaining higher levels of TP that could trigger more eutrophication events in the future. Trophic state index deviation (TSID) is a multidimensional approach and holds an essential place in the lake and reservoir ecosystems’ structure and management. As it designates the working of aquatic plants that construct the foundation of the aquatic food chain, it advocates for the inclusive role of nutrients used to measure the trophic status [50]. Phosphorus does not emerge as a single limiting nutrient in these conditions. However, detailed bioassay investigations could provide some reliable estimates of the specific nutrient levels. These findings are consistent with previous studies performed in South Korean reservoirs [2,66].

### 3.7. Seasonal Trend Analyses of Water Chemistry

The seasonal MKT provided significant insights into the existing trends in crucial regulatory factors in the Soyang reservoir. The parameters included TN, TP, Chl-a, BOD, COD, EC, TSS, and Chl-a (Table 3). In the Soyang reservoir, WT, DO, COD, TSS, TP, and Chl-a displayed decreasing trends in the ambient water. On the contrary, pH, COD, EC, and TN were observed to be increasing at a slow pace. The increasing tendency of COD was affected by nondegradable matters. An increase in EC can indirectly determine the increase of ionic substances. These trends led to inferring that the TN and TP opposite trends implied that nitrogen and phosphorus were present in a state that was not supportive of the future eutrophication in the Soyang reservoir. It is further strengthened by the negative trend in TSS and EC. However, positive tendencies of COD and pH allude to the increasing influx of the industrial and agricultural runoff. The MKT outcomes stress the proficient management of the anthropogenic activities in the water basin of the Soyang reservoir. It is mention-worthy that the trends shown by MKT do not inherently indicate these trends will maintain in the coming years, because these trends are contingent on the magnitude of input variables, as certain variables are subject to anthropogenic influences [25,67].

### 3.8. Evaluation of Water Pollution Status

The zonal water quality status assessed by the application of modified WPI indicated the *Lz* was in excellent water quality status while *Tz* and *Rz* displayed good water quality status on the basis of salient water chemistry parameters (TN, TP, TN:TP ambient ratios, TSS, BOD, EC, and Chl-a). The details of the ascribed scoring criteria and their outcomes are shown in Table 4. Based on a total of seven metrics representing the most influential water quality factors, WPI has been recently used to designate the water quality status of freshwater resources. These water chemistry parameters could be used as indicators of water quality degradations if they displayed palpable changes in their values under the impact of anthropogenic activities [4,68]. These parameters represent the nutrient regime (TN, TP, TN:TP ratios), organic matter (BOD), dissolved and suspended ionic contents (TSS and EC), and Chl-a as the indicator of primary production status. All these parameters are of utmost importance to the maintenance of environmental water quality. The detailed breakdown of the obtained water quality scores indicated that the water quality in the *Lz* remained oligotrophic with respect to TN (1.50 ± 0.29), TP (16.5 ± 23.1) and TN:TP ratios (158.4 ± 110.3). However, the nutrient regime suggested that the water quality in TZ and Rz remained mesotrophic (1.5–3.0 mg/L) with respect to TN and oligotrophic with respect to TP (<30 µg/L) and TN:TP (<50). The organic matter and ionic contents (TSS and EC) indicated no differences among the lake zones during the whole study. However, the obtained scores and mean values of Chl-a mostly approximated with those of TN, TP, and their ambient ratios in the *Rz* and *Tz*. The lowest mean level of Chl-a (2.9 ± 3.29) was noticed in the *Lz,* supporting the notion settled by the presence of loads of TN in the same zone. The total scores guided classifying the lake zones based on water pollution status ascribed according to the given criterion of WPI. The *Rz* and *Tz* indicated good water quality status (27 each) while *Lz* displayed excellent (31) quality status. Overall, the outcomes indicated increasing trends in the levels of nitrogen and phosphorus from the *Lz* to *Rz* in Soyang reservoir. Substantial increments in the solid contents indicated the speeding up of sedimentation rate in the Rz, which could lead to decreased reservoir capacity in the future [69,70]. Recently conducted studies showed TN:TP ambient ratios as indirectly used physiochemical water quality indicators that provide useful hints at the nutrient limitations for primary production in terms of Chl-a [35]. The declining TN:TP ambient ratios allude to the increasing nutrient pollution that could ultimately result in eutrophication of lakes and reservoirs [69]. Several researches have supported this type of chemical health degradation in lakes and reservoirs [4,70,71,72].

## 4. Conclusions

In conclusion, the chemical water quality evaluation and comparisons on the spatial and seasonal levels between Chl-a and nutrient-contributing factors displayed heterogenic trends in the reservoir water quality status. The spatial and seasonal heterogeneities in the prominent water chemistry factors and nutrients alluded to the dominant impact of monsoon regime in the reservoir watershed as well as in the three zones of the reservoir. The results illustrated weak positive relations between the Chl-a and TP. The influence of intensive rainfall prevailed as the most crucial factor and created alternative flood and drought events in the reservoir watershed that conspicuously impacted the salient water quality parameters. The trophic status deviations presented a mixed trophic state, except zooplankton grazing. The flood years indicated phosphorus limitation, while drought years alluded to the non-algal light limitation in the Soyang reservoir. TP and Chl-a showed a decreasing trend under the Mann–Kendal trend analysis. The water pollution index designated the lacustrine zone with excellent water quality, indicating, thereby, the least impact of anthropogenic activities on nutrient regime and solid contents. The productivity of Chl-a approximated the zonal trends indicated by the nutrient-contributing factors from lacustrine to riverine zones in the reservoir. Overall, the impact of intense monsoon rainfall remained crucial in water quality transformation, nutrient enrichment, drought and flood dynamics, longitudinal and zonal disparities, and trend detection. Our research recommends more in-depth investigations into the drought and flood dynamics and their impacts on the water quality fluctuations.

## Figures and Tables

**Table 1:** Summary statistics of water chemistry parameters in the three zones of the Soyang reservoir studied during 1992–2013.

Water Quality Attributes	Lacustrine Zone (L_z_)		Transition Zone (T_z_)		Riverine Zone (R_z_)	
	Min-Max	Mean ± Stand Dev	Min-Max	Mean ± Stand Dev	Min-Max	Mean ± Stand Dev
pH	5.4–9.4	7.12 ± 0.57	5.2–9.5	7.23 ± 0.55	5–9.2	7.37 ± 0.57
WT	4–28	9.54 ± 5.83	1–28	12.03 ± 6.61	0–30	13.92 ± 7.75
DO	1.1–13.6	8.86 ± 2.14	0.8–89.6	9.4 ± 5	3.5–17.7	9.87 ± 2.06
BOD	0.3–2.5	1.04 ± 0.3	0.1–2.7	1.19 ± 0.27	0.6–2.6	1.27 ± 0.32
COD	1.1–7.6	2.16 ± 0.62	1.3–12.8	2.27 ± 0.61	1.1–5.3	2.33 ± 0.56
TN	0.73–2.9	1.50 ± 0.29	0.71–4.18	1.74 ± 0.44	1.01–3.65	1.81 ± 0.47
NH_4_-N	0–0.34	0.02 ± 0.03	0–2.05	0.03 ± 0.07	0–0.14	0.03 ± 0.02
TDN	0.63–2.02	1.4 ± 0.23	0.77–3.64	1.57 ± 0.38	0.85–3.42	1.58 ± 0.43
NO_3_-N	0.09–3.38	1.2 ± 0.25	0.02–845	2.25 ± 27.8	0.63–2.7	1.36 ± 0.35
TP	1–237	16.5 ± 23.1	2–386	19.8 ± 23.2	2–156	20.7 ± 16.7
TDP	0–1.73	0.02 ± 0.15	0–1.89	0.02 ± 0.16	0–0.04	0.01 ± 0.01
PO_4_-P	0–0.06	0 ± 0.01	0–0.11	0 ± 0.01	0–0.03	0 ± 0
TN:TP	6.35–1239	158.4 ± 110.3	5.72–663	129.4 ± 80.6	15.9–558	120.4 ± 74.6
SD	3.4–100	43.8 ± 32.5	0.2–60	17.4 ± 15.3	0.2–45	6 ± 5.69
TSS	0–168	4.58 ± 13.7	0.1–216	6.16 ± 16.2	0.2–179	7.38 ± 18.9
EC	41–432	68.37 ± 23.84	10–127	70.16 ± 14.75	0–128	70.88 ± 16.25
TCB	0–350	48.13 ± 59.01	0–1600	112.5 ± 211.1	2–2400	170.6 ± 305.6
Chl-a	0–24.8	2.9 ± 3.29	0–40.1	4.61 ± 4.98	0–85.8	4.55 ± 7.52

Note: Min = Minimum; Max = Maximum; Stand Dev = Standard deviation; SD = Secchi disk (m); DO = Dissolved oxygen (mg/L); BOD = Biological oxygen demand (mg/L); COD = Chemical oxygen demand (mg/L); TSS = Total suspended solids (mg/L); TN = Total nitrogen (mg/L); NH_4_-N = Ammonia nitrogen (mg/L); NO_3_-N = Nitrate nitrogen (mg/L); TP = Total phosphorus (µg/L); TN:TP = Ambient ratios of TN and TP; WT = Water temperature (°C); EC = Electrical conductivity (mS/cm); TCB = Total coliform bacteria, TDN = Total dissolved nitrogen (mg/L); TDP = Total dissolved phosphorus (mg/L); PO_4_-P = Phosphate phosphorus (mg/L); Chl-a = Chlorophyll-a (µg/L).

**Table 2:** Trophic state index (TSI) during pre-monsoon, monsoon, and post-monsoon in the Soyang reservoir.

Season	TSI (TN)	TSI (TP)	TSI (Chl-a)
Premonsoon	61	43	40
Monsoon	61	52	48
Postmonsoon	61	47	46

Note: TN: total nitrogen; TP: total phosphorus; Chl-a: chlorophyll.

**Table 3:** Seasonal Mann–Kendal trend analyses in Soyang reservoir during 1992–2013.

Parameters	Tau Correlation	*S*	*Z*	*p*-value	Empirical Model	Trend Analysis
WT	−0.158	−411	−3.517	0.0416	WT = 12.07 − 0.5000E-01 × T	−
pH	0.238	620	5.304	0.0092	pH = 6.958 + 0.1667E-01 × T	+
DO	−0.036	−94	−0.793	0.5107	DO = 9.372 − 0.6250E-02 × T	−
BOD	−0.139	−362	−3.104	0.2543	BOD = 1.246 − 0.8333E-02 × T	−
COD	0.287	748	6.400	0.0048	COD = 1.913 + 0.2500E-01 × T	+
TSS	−0.046	−121	−1.023	0.6476	TSS = 2.680 − 0.2000E-01 × T	−
EC	0.344	897	7.642	0.0026	EC = 58.33 + 0.6667 × T	+
TN	0.167	436	3.702	0.1673	TN = 1.411 + 0.1131E-01 × T	+
TP	−0.058	−152	−1.289	0.5190	TP = 15.82 − 0.7143E-01 × T	−
Chl-*a*	−0.131	−342	−2.906	0.1361	Chl-*a* = 3.200 − 0.5000E-01 × T	−

Note: S = Statistic, Z = Standardized variable, T = Time (minutes), WT = Water temperature (°C), DO = Dissolved oxygen (mg/L); BOD = Biological oxygen demand (mg/L); COD = Chemical oxygen demand (mg/L); TSS = Total suspended solids (mg/L); EC = Electrical conductivity (mS/cm); TN = Total nitrogen (mg/LTP = Total phosphorus (µg/L); Chl-a = Chlorophyll-a (µg/L).

**Table 4:** Chemical health status of three zones of Lake Soyang based on modified water pollution index (WPI) during 1992–2013.

**Category**	**Model Metrics (M)**	**Scoring Criteria**			**Mean ± Standard Deviation** **Scores**		
		5	3	1	Rz	Tz	Lz
Nutrient Regime	M_1_: Total Nitrogen (mg/L)	<1.5	1.5–3.0	>3	1.81 ± 0.47 (3)	1.74 ± 0.44 (3)	1.50 ± 0.29 (5)
	M_2_: Total Phosphorus (µg/L)	<30	30–100	>100	20.7 ± 16.7 (5)	19.8 ± 23.2 (5)	16.5 ± 23.1 (5)
	M_3_: TN:TP ratio	>50	20–50	<20	120.4 ± 74.6 (5)	129.4 ± 80.6 (5)	158.4 ± 110.3 (5)
Organic Matter	M_4_: Biological Oxygen Demand (mg/L)	<1	1–2.5	>2.5	1.27 ± 0.32 (3)	1.19 ± 0.27 (3)	1.04 ± 0.3 (3)
Ionic Contents and Solids	M_5_: Total Suspended Solid (mg/L)	<4	4–10	>10	7.38 ± 18.9 (3)	6.16 ± 16.2 (3)	4.58 ± 13.7 (3)
	M_6_: Electrical Conductivity (µS/cm)	<180	180–300	>300	70.88 ± 16.25 (5)	70.16 ± 14.75 (5)	68.37 ± 23.84 (5)
Primary Production Indicator	M_7_: Sestonic Chlorophyll (µg/L)	<3	3–10	>10	4.55 ± 7.52 (3)	4.61 ± 4.98 (3)	2.9 ± 3.29 (5)
	Final Scores of WPI				27	27	31
	Water Quality				Good	Good	Excellent

Rz = Riverine zone; Tz = Transition zone; Lz = Lacustrine zone.

## Supplementary Materials

The following are available online at https://www.mdpi.com/1660-4601/17/9/3139/s1, Table S1: Detailed location of study sites in the Soyang reservoir.
