# Supplementary material for: Multiyear Links between Water Chemistry, Algal Chlorophyll, Drought-Flood Regime, and Nutrient Enrichment in a Morphologically Complex Reservoir
Source: Int J Environ Res Public Health. 2020 Apr 30;17(9):3139. doi: 10.3390/ijerph17093139 (PMC7246998; doi:10.3390/ijerph17093139)
Supplement: Supplementary file 1 [file ijerph-17-03139-s001.pdf]

Supplementary Material

Table S1. Detailed location of study sites in the Soyang reservoir.

Site	Address	Coordinates
Soyang Dam 1	Cheonjeon-ri, Sinbuk-eup, Chuncheon-si, Gangwon-do	127°49′16.05″E 37°56′40.92″N
Soyang Dam 2	Ohang-ri, Buksan-myeon, Chuncheon-si, Gangwon-do	127°54′38.68″E 37°57′42.95″N
Soyang Dam 3	Seokhyeon-ri, Yanggu-eup, Yanggu-gun, Gangwon-do	127°57′45.88″E 38°02′05.45″N
Soyang Dam 4	Bupyeong-ri. Nam-myeon, Inje-gun, Gangwon-do	128°05′58.51″E 38°00′22.73″N
Soyang Dam 5	Sangsunae-ri, Nam-myeon, Inje-gun, Gangwon-do	128°03′33.25″E 37°59′28.52″N
